# Demonstration of a terahertz pure vector beam by tailoring geometric phase

**DOI:** 10.1038/s41598-018-26964-7

**Published:** 2018-06-06

**Authors:** Toshitaka Wakayama, Takeshi Higashiguchi, Kazuyuki Sakaue, Masakazu Washio, Yukitoshi Otani

**Affiliations:** 1 School of Clinical Engineering, Faculty of Health and Medical Care, Saitama Medical University, 1397-1, Yamane, Hidaka, Saitama 350-1241 Japan; 2 Department of Electrical and Electronic Engineering, Faculty of Engineering, Utsunomiya University, 7-1-2, Yoto, Utsunomiya, Tochigi 321-8585 Japan; 3 Center for Optical Research & Education (CORE), Utsunomiya University, 7-1-2, Yoto, Utsunomiya, Tochigi 321-8585 Japan; 4 Waseda Institute for Advanced Study, Waseda University, 3-4-1, Okubo, Shinjuku, Tokyo 169-8555 Japan; 5 Research Institute for Science and Engineering, Waseda University, 3-4-1, Okubo, Shinjuku, Tokyo 169-8555 Japan; 6 Department of Optical Engineering, Faculty of Engineering, Utsunomiya University, 7-1-2, Yoto, Utsunomiya, Tochigi 321-8585 Japan

## Abstract

We demonstrate the creation of a vector beam by tailoring geometric phase of left- and right- circularly polarized beams. Such a vector beam with a uniform phase has not been demonstrated before because a vortex phase remains in the beam. We focus on vortex phase cancellation to generate vector beams in terahertz regions, and measure the geometric phase of the beam and its spatial distribution of polarization. We conduct proof-of-principle experiments for producing a vector beam with radial polarization and uniform phase at 0.36 THz. We determine the vortex phase of the vector beam to be below 4%, thus highlighting the extendibility and availability of the proposed concept to the super broadband spectral region from ultraviolet to terahertz. The extended range of our proposed techniques could lead to breakthroughs in the fields of microscopy, chiral nano-materials, and quantum information science.

## Introduction

Vector beams, which tailor spatial distribution of polarization and phase, have the potential to lead to cutting-edge developments in the fields laser physics^1^, biological imaging^2,3^, light-matter interaction^4,5^, next-generation quantum communication^6^, electron acceleration^7,8^, and astronomy^9^. Pure vector beams, which form the basis of vector beams, such as radially and azimuthally polarized beams^10–12^, produce a longitudinal electric field^11–13^ and an optical Möbius strip^14^. Researchers have also shown that by adjusting the intensity distribution of a pure vector beam with radial polarization, one can control the three-dimensional distribution of the electric field^15^. Moreover, the ability to focus the electric field by a pure vector beam enables a beam spot to be sharpened to sizes smaller than the diffraction limit – an ability that cannot be achieved with linearly polarized beams^13^. Because of these optical properties, pure vector beams are expected to enable super-resolution in microscopy and laser processing^11,13,16^. However, the super-resolution property of pure vector beams is destroyed by the geometric phase induced by cyclic polarization changes^17–19^.

When a generated beam is radially polarized, but its phase is spirally distributed as vortex phase, the beam is referred to as a radially polarized optical vortex^20,21^. Propagation of a beam containing a radially polarized optical vortex is different from that of a radially polarized beam^12^. Such a radially polarized optical vortex shows complex polarization dynamics because its polarization and phase are spatially distributed in three-dimensional free space. For this reason, vortex phase cancellation by use of an optical vortex with opposite vortex phase has been proposed as a method of creating pure vector beams^3,22^. This approach, however, is limited to experimental setups with complex optical alignments and low optical material throughput using polarization elements. To be best of our knowledge, radial polarization converters, which enable conversion to be the pure vector beams, have been proposed for use in a micro-structured photoconductive antenna^23^, a cone lens^24^, laser resonances^1^, liquid crystal modulators^6,12^, sub-wavelength structures^15,17^, nonlinear crystals^25^, wave plates of form birefringence fabricated by three-dimensional printing^26^, and meta-materials^2,27^ in several spectral regions from visible to terahertz. Our group also illustrated an achromatic axially-symmetric wave plate based on internal reflections and a generation of vector beams in the visible^22,28^, middle infrared^18^, and terahertz^29^ spectral regions. To verify details of the pure vector beam generated by use of those radial polarization converters, one must verify that the generated beam removes the vortex phase that can result from geometric phase. Although the vortex phase must be reduced in applications of pure vector beams, few studies have rigorously determined the spatial distribution of phase and its polarization of generated vector beams^2,3,7,8,15,17,23^, having observed only intensity distributions transmitted through a polarizer. The radial polarization converters used in these studies, moreover, are limited to narrow wavelength bands because broadband vortex phase cancellation has not been previously demonstrated.

In this paper, we specifically show a pure vector beam that eliminates the vortex phase by tailoring geometric phase at 0.36 THz. Our proposed concept of vortex phase cancellation considers symmetry of the polarization of the light and asymmetry of the polarization converter. The asymmetry of the radial polarization converter enables one to remove vortex phase in generated vector beam. Geometric phase, which is induced by the spatial distribution of polarization in the vector beam, determines whether the generated vector beam has vortex phase. For this reason, we also propose single shot determination of polarization distribution in the generated vector beam. Our work provides insights that could lead to new frontiers in highly important scientific fields involving singular optics^30^ and quantum communications^31,32^ using pure vector beams, which are expected to represent a breakthrough for surpassing the diffraction limit, high-density transmission of signals, and generating longitudinal electric fields by light. We focus on the proof-of-principle demonstration of a terahertz (THz) pure vector beam by tailoring the geometric phase. In our demonstration, we also show that our concept is not limited to the THz region only and enables us to extend this approach to different spectral regions.

## Results

### Pure vector beams with radial polarization

**Figure 1:**
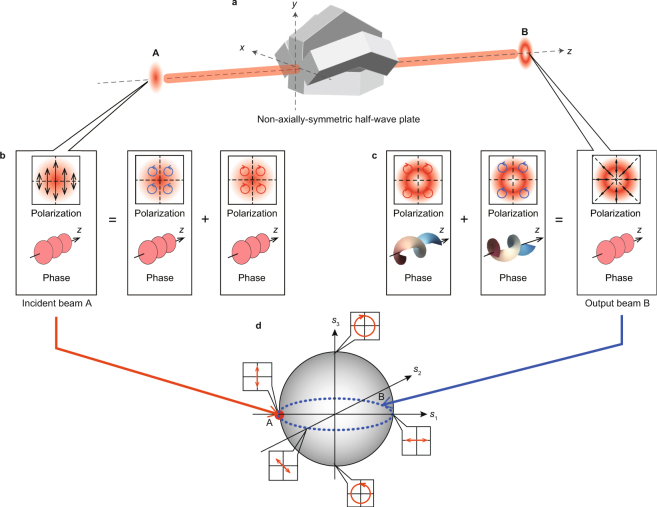
Concept of vortex phase cancellation in vector beams. (**a**) The non-axially-symmetric half–wave plate converts an incident 90° linearly polarized beam, with phase illustrated by “A”, into an output radially polarized beam with phase illustrated by “B”. We note that the intensity distribution of the incident beam “A” is a Gaussian profile while the output beams form doughnut-like profiles. (**b**) Linear polarization can be considered as a sum of left and right circular polarizations with uniform phase. (**c**) After passing through a non-axially-symmetric half-wave plate, the pair of left and right circular polarizations is converted into opposite circular polarizations having vortex phase. The output beam “B” becomes radially polarized with uniform phase because optical vortices have eliminated the beam’s vortex phases. (**d**) Changes of polarization on the vector beam after passing through the non-axially-symmetric half–wave plate are illustrated on a Poincaré sphere. Incident polarization is located at point “A” on equator. After passing through the non-axially-symmetric half-wave plate, the linear polarization “A” is transformed the polarization “B” illustrated by the equator of the Poincaré sphere. Because of the geometric phase produced by cyclic changes of the polarization on a Poincaré sphere, the phases at the point “B” are everywhere zero.

We theoretically investigate the spatial distribution of polarization and vortex phase of vector beams. The key concept for reduction of vortex phase in vector beams is the cancellation of vortex phase by tailoring left- and right-hand circularly polarized optical vortices. This cancellation is achieved from the relationship between symmetry of polarized light and a rotational asymmetry of a polarization converter (see Fig. 1). In the figure, the incident beam, which has linear polarization described at “A”, is converted into output beam “B” via radial polarization converters as a non-axially-symmetric half-wave plate (Fig. 1a). The incident linear polarization consists of a pair of left and right circular polarizations with uniform phase (Fig. 1b). Considering polarization conversion of the incident circular polarizations by the non-axially-symmetric half-wave plate, left and right circular input polarizations with uniform phase are reversed to give right and left circular output polarization. The phase distributions of the circularly polarized beams are spirally distributed with vortex phase because of the non-axially-symmetric half-wave plate (Fig. 1c). After the circularly polarized vortex beams are combined, the output beam illustrated at “B” becomes a radially polarized beam with uniform phase because the vortex phase is eliminated.

To verify the abovementioned concept, we estimate the phase of the generated vector beam by calculating the geometric phase induced by cyclic changes of the polarization^33^. The changes of the generated beam’s polarization state are sketched on a Poincaré sphere (Fig. 1d). When the incident linear polarization (red circle) is converted into radial polarization, changes of the polarization state on the Poincaré sphere is described on the equator (blue dashed line) of the Poincaré sphere because the radial polarization has linear polarizations corresponding with the angle *θ*. The area closed by cyclic changes of the polarization on the Poincaré sphere is zero. As a result, the generated beam becomes a pure vector beam having radial polarization and uniform phase. For linear polarization, which consists of left and right circular polarization, the non-axially-symmetric half-wave plate enables cancellation of the vortex phase.

**Figure 2:**
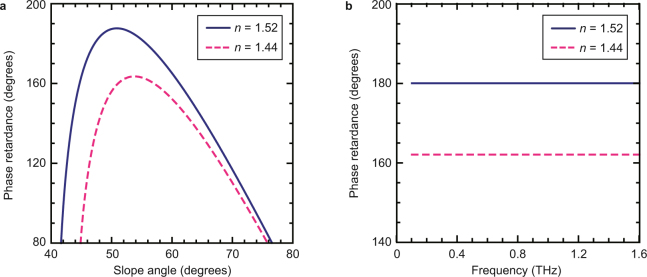
Phase retardance by refractive indices. (**a**) Relationship between slope angle of non-axially-symmetric half-wave plate and phase retardance. An incident linearly polarized beam generates phase retardance after reflection within a Fresnel rhomb with refractive index *n* and slope angle *β*. Phase retardance is related to the slope angles and refractive indices *n* = 1.52 (solid, blue) of high-density polyethylene (HDPE) and *n* = 1.44 (dashed, red) polytetrafluoroethylene (PTFE). (**b**) Frequency dependences of phase retardance are described with *n* = 1.52 (solid, blue) and *n* = 1.44 (dashed, red), respectively. HDPE and PTFE enable the use of THz frequency regions. The slope angles of HDPE and PTFE are set to 55° due to the non-axially-symmetric half-wave plate and the axially-symmetric wave plate, respectively.

We calculate the phase retardance generated by internal reflections within the non-axially-symmetric half–wave plate. We investigate the phase retardance along the slope angle of the non-axially-symmetric half-wave plate (Fig. 2a) as well as THz frequency dependence of the phase retardance (Fig. 2b). Phase retardance is related to the slope angles and refractive indices *n* = 1.52 (solid, blue) of high-density polyethylene (HDPE) and *n* = 1.44 (dashed, red) polytetrafluoroethylene (PTFE). (see the subsection “Internal Fresnel Reflections” in the Methods section) We estimate that total internal reflection produces achromatic phase retardance in the frequency region from 0.1 to 1.6 THz (for more information, refer the subsection non-axially-symmetric half-wave plate, “Jones calculus” in the Method sections, and Supplementary Note 1 and 2). HDPE generates a retardance of 180° due to four internal reflections at the angle of 55° due to create the radially polarized beam with uniform phase.

### Beam profile of the vector beam

**Figure 3:**
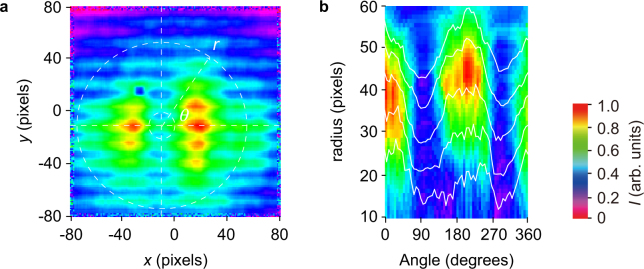
Experimental results for generating a vector beam at 0.36 THz. The intensity distribution across a vector beam viewed through a polarizer, used to illustrate the spatial distribution of polarization. (**a**) After a vertically polarized beam with Stokes parameters of (1, −1, 0, 0)^T^ is passed through a non-axially-symmetric half–wave plate and a wire-grid polarizer oriented at 0°, the intensity distribution represented by rectangular coordinates is captured by a pyroelectric array camera. The obtained image shows that the vector beam was successfully converted. However, details of the spatial distribution of the polarization are not easily captured by the image. (**b**) The same image shown in polar coordinates. Intensity profiles at radius *r* = 10, 20, 30, 40, and 50 lines are described by the white lines. The image shown in **b** is available for the analysis of the spatial distribution of the polarization on the vector beam.

In our proof-of-principle experiments, we constructed an experimental setup consisting of a vector beam polarization converter and polarization analyzer (see the subsection “Experimental setup” in the Methods section and Supplementary Note 3). We obtain an image (Fig. 3a) using a pyroelectric camera after light beam passes through an axially-symmetric wave plate and a THz polarizer. In general, such images likely indicate a radially polarized beam because the transparency angle of the THz polarizer was set at 0°. Details of the spatial distribution of polarization of the generated vector beam, however, are difficult to observe in the image of intensity distribution. To resolve this issue, we develop a full analytical theory to determine the spatial distribution of polarization on the vector beam. For effective analysis of the vector beam, the image was transformed from Cartesian *x*-*y* coordinates to polar coordinates as shown in Fig. 3b. From Fig. 3a and b, we can evaluate the polarization distribution of the vector beam because the intensity distribution is modulated along the angle *θ*. The intensity distribution enables single shot determination of the polarization on the vector beam^29^.

### Spatial distribution of polarization on the vector beam

**Figure 4:**
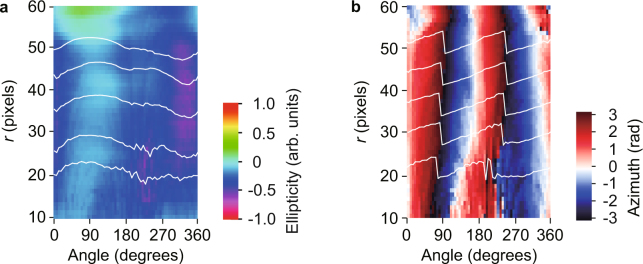
Spatial distribution of polarization for vector beams. Spatial distribution of the polarization ellipticity (**a**) and azimuth (**b**) with respect to the azimuth angle *θ* within the beam. Ellipsometric parameters show that the vector beam is radially polarized because the ellipticity is almost zero and its azimuth varies linearly with the angle *θ*. However, the phase of the vector beam was not measured.

Rather than performing a conventional polarization analysis, we perform a single-shot determination of the polarization of the vector beam without the effects of fluctuating light sources and the rotation of polarization elements (see the subsection “Single-shot determination of polarization on vector beams” in the Methods section, and Supplementary Note 4). Using the image shown in Fig. 3b, we determine the two-dimensional distribution of the polarization, such as ellipticity (Fig. 4a) and azimuth angle (Fig. 4b). The measured ellipticity and azimuth are consistent with the theory of pure vector beams with radial polarization because the ellipticity is nearly zero and the azimuth varies linearly. For this case, the vector beam is radially polarized. However, we cannot determine whether the vector beam successfully removes the vortex phase because its phase distribution was not known.

**Figure 5:**
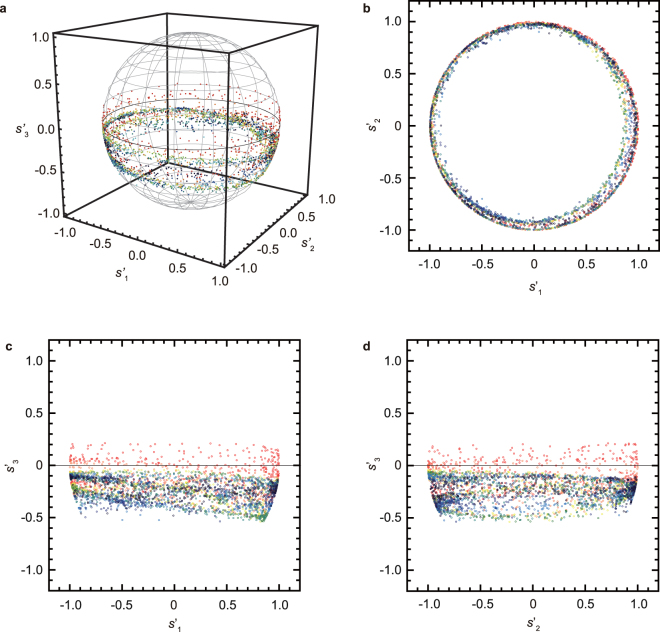
Details of the generated vector beam. The vector beam’s normalized Stokes parameters are depicted by colour-coded plots of red (*r* = 10–19 pixels), orange (*r* = 20–29 pixels), yellow (*r* = 30–39 pixels), green (*r* = 40–49 pixels), and blue (*r* = 50–59 pixels) on a Poincaré sphere (**a**). Subfigures (**b**–**d**) show planar projections of the data onto planes *s*_1_–*s*_2_ (**b**), *s*_1_–*s*_3_ (**c**), *s*_2_–*s*_3_ (**d**). Even though the generated vector beam has become radially polarized, Stokes parameter *s*_3_ remains close to zero. The polarization on the vector beam slightly becomes elliptical from (**c**) and (**d**) due to a small shift of slope angle on the non-axially-symmetric half-wave plate — a product of limitations of THz beam collimation by the first lens in the optical system (see “Experimental setup” in Method).

To elucidate whether the generated beam reduces the vortex phase, we investigate the phase as well as the polarization of the vector beam. Simultaneously determining both properties of the vector beam is difficult because an identical setup is required for a wavefront sensor and an interferometer. Stokes parameters determined by single-shot determination of the spatial distribution of the polarization on the vector beam are normalized to obtain the geometric phase. The Stokes parameters are distributed on the Poincaré sphere (Fig. 5a), a *s*′_1_-*s*′_2_ plane projection (Fig. 5b), a *s*′_1_-*s*′_3_ plane projection (Fig. 5c), and a *s*′_2_-*s*′_3_ plane projection (Fig. 5d). Evaluating the Poincaré representation, we confirm the changes of the polarization of the vector beam corresponded to the polarization distribution on the Poincaré sphere shown in Fig. 1d (for more information, see Supplementary Note 4).

### Geometric phase of the vector beam with radial polarization

**Figure 6:**
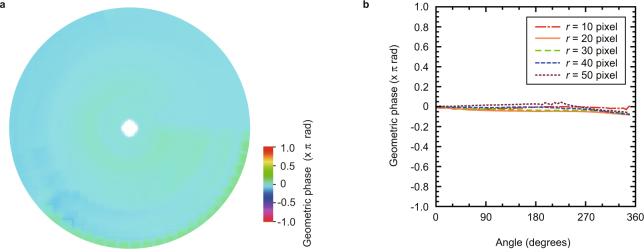
Details of the measured geometric phase. A requirement for a pure vector beam with radial polarization is a uniform phase and not a vortex phase around the beam. To demonstrate that the generated beam becomes the pure vector beam, the geometric phase is calculated from area enclosed by Stokes parameters on a Poincaré sphere shown in Fig. 5. The geometric phase is uniformly distributed around the beam (**a**). The geometric phase is plotted as the function of the angle *θ*, which reaches the maximum of −0.23 rad at *r* = 20 pixels and *θ* = 2π rad (**b**). These results provide evidence that the vortex phase has been removed because the determined geometric phase is low compared with a radially polarized optical vortex with a geometric phase of 2π rad.

To demonstrate the reduction of the vortex phase, we evaluate the geometric phase of the experimentally generated beam. The two-dimensional distribution (Fig. 6a) and the radius dependence (Fig. 6b) of the geometric phase are calculated from the cyclic changes of the polarization on the Poincaré sphere (see the subsection “Determination of the geometric phase” in the Methods section, and Supplementary Note 5). The average and maximum of the geometric phase were −0.01 rad and −0.23 rad, respectively. These values of the geometric phase obtained by our result were much smaller than ±6.28 rad, which is that of the radially polarized optical vortex. As a result, the phase of the vector beam was uniformly distributed because the vortex phase of the vector beam eliminated 96%. To the best of our knowledge, this work provides the first demonstration of the tailoring of the geometric phase due to a pure vector beam. This approach, however, is limited by the pulse width of the beam because of the influence of the group velocity dispersion.

## Discussion

We showed a pure vector beam by tailoring geometric phase in 0.36 THz. As shown in Fig. 2, the non-axially-symmetric half-wave plate is also calculated achromatic properties of phase retardance. Such achromatic phase retardance is resulted by frequency dependence of refractive index as shown in Fig. 2b. Our experimental results containing not only the polarization distribution but also the geometric phase agreed well with calculated results. In our previous study, we have also shown achromatic properties of vectorial vortex beams using an axially-symmetric wave plate at the frequencies of 0.16 and 0.36 THz^29^. For this reason, our proposed device is possible to generate achromatic pure vector beam in THz regions.

**Figure 7:**
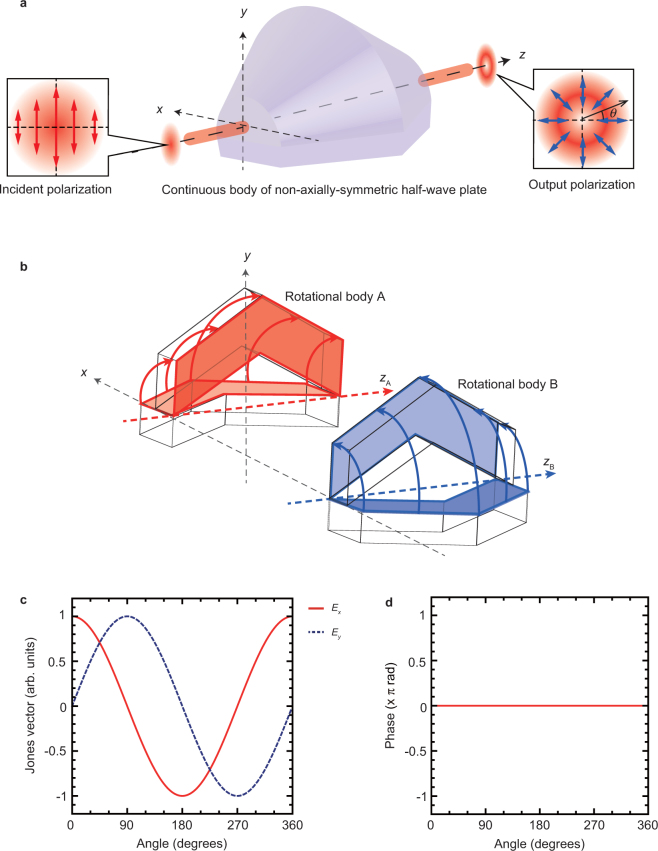
Continuous body of the non-axially-symmetric half-wave plate. Relationship *D*/*λ* between an aperture diameter *D* and long wavelength *λ* of THz regions was 50 as shown in Table 1. This helps to achieve pure vector beam with radial polarization. However, precisely fabricating an optical element as shown in Fig. 1a in the ultraviolet, visible, and infrared regions is difficult because the optical configuration is complicated. (**a**) To address this drawback, we propose a non-axially-symmetric half-wave plate with a continuous body. (**b**) The non-axially-symmetric half–wave plate consists of two rotational bodies “A” and “B” of Fresnel rhomb. We numerically simulated Jones vectors of [*E*_*x*_(*θ*), *E*_*y*_(*θ*)]^T^ of the vector beam along the angle *θ* after passing through the continuous non-axially-symmetric half–wave plate. (**c**) Jones vectors of *E*_*x*_(*θ*) (red solid line) and *E*_*y*_(*θ*) (blue dashed line) are shown, respectively. The spatial distribution of the polarization on the vector beam is radially polarized because Jones vectors show [*E*_*x*_(0°), *E*_*y*_(0°)]^T^ = (1, 0)^T^, [*E*_*x*_(90°), *E*_*y*_(90°)]^T^ = (0, 1)^T^, [*E*_*x*_(180°), *E*_*y*_(180°)]^T^ = (−1, 0)^T^, and [*E*_*x*_(270°), *E*_*y*_(270°)]^T^ = (0, −1)^T^. (**d**) Phase of the vector beam is uniformly distributed. According to these results, a pure vector beam with radial polarization can be generated by the continuous non-axially-symmetric half–wave plate proposed in (**a**). This optical element with the continuous body is suitable for fabrication in the ultraviolet, visible, infrared, and THz spectral regions.

The range of our proposed approach extends to all spectral regions from ultraviolet to terahertz. Table 1 shows the possible materials for the use in the non-axially-symmetric half-wave plate for the different spectral regions. SiO_2_, BK7, ZnSe, PTFE, HDPE, and Tsurupica enable fabrication of the segmented rhomb in each spectral region. However, for the segmented non-axially-symmetric half–wave plate shown in Fig. 1a, practical wave plate materials are limited for ultraviolet, visible, and infrared regions because of issues associated with the complex assembly and high processing accuracy. To overcome this drawback, we propose constructing a continuous non-axially-symmetric half–wave plate body as shown in Fig. 7a. In previous study, the continuous body has realized from form birefringence fabricated by three-dimensional printer^26^. However, the device is limited by the frequency of the THz light source because of frequency dependence of form birefringence. On the other hand, our proposed continuous body provides the generation of achromatic pure vector beams by the internal Fresnel reflection. Our continuous non-axially-symmetric half–wave plate body consists of two rotational bodies “A” and “B” (Fig. 7b). These optical elements are formed by as the volume generated by rotating a Fresnel rhomb through an angle of 90° along the axes *z*_A_ and *z*_B_, respectively. After passing through the continuous non-axially-symmetric half–wave plate, four internal reflections generate the vector beam. We numerically calculate the spatial distribution of polarization of [*E*_*x*_(*θ*), *E*_*y*_(*θ*)]^T^ of the vector beam via Jones calculus. The Jones vectors of *E*_*x*_(*θ*) (red solid line) and *E*_*y*_(*θ*) (blue dashed line) are shown in Fig. 7c. The spatial distribution of polarization on the vector beam is radially polarized because the typical Jones vectors were represented by [*E*_*x*_(0°), *E*_*y*_(0°)]^T^ = (1, 0)^T^, [*E*_*x*_(90°), *E*_*y*_(90°)]^T^ = (0, 1)^T^, [*E*_*x*_(180°), *E*_*y*_(180°)]^T^ = (−1, 0)^T^, and [*E*_*x*_(270°), *E*_*y*_(270°)]^T^ = (0, −1)^T^. In addition to the spatial distribution of the polarization, the phase of the vector beam is uniformly distributed as shown in Fig. 7d. This confirms that it should be possible to generate a pure vector beam with radial polarization via the proposed continuous non-axially-symmetric half–wave plate of Fig. 7a. In addition to radial polarization, azimuthally polarized beam, which is another of pure vector beams, also enables beam generation by changing the polarization angle at 0° of the incident linear polarization. The continuous body of this non-axially-symmetric half–wave plate is suitable for use in the superbroadband spectral region from ultraviolet to THz as shown in Table 1. We expect to create the continuous body because three-dimensional printing of transparent fused silica glass enables the construction of complex shapes on optical elements^34^.

**Table 1:** Possible configurations for a continuous non-axially-symmetric half–wave plate.

Spectral regions	Wavelength	Material	Slope angle (degrees)	*D*/*λ*
Ultraviolet	200–410 nm	SiO_2_	50°	10^5^
Visible	400–800 nm	SiO_2_, BK7	52°, 55°	5 × 10^4^
Infrared	532 nm–2 μm	ZnSe	65°	2 × 10^4^
Terahertz	200 μm–3 mm	HDFE, PTFE, Tsurupica	55°	50

*D* and *λ* indicate the aperture diameter of optical element and emerging wavelength, respectively.

SiO_2_ in the ultraviolet region provides a retardance of 180° due to four internal reflections at a the slope angle of 50°.

SiO_2_ and BK7 on visible region give a retardance of 180° due to eight and four internal reflections at a slope angle of 52° and 55°, respectively.

ZnSe yeilds a retardance of 180° due to four internal reflections at a slope angle of 65°.

PTFE gives a retardance of 162° due to four internal reflections at a slope angle of 55°.

HDPE, and Tsurupica also generate a retardance of 180° due to four internal reflections at a the slope angle of 55°, respectively.

As compared with other spectral regions, *D*/*λ* in the THz region is small. However, the aperture size of optical element is large enough to employ a THz light source.

In conclusion, we have proposed and demonstrated a pure vector beam without vortex phase by tailoring the geometric phase of the beam. This paper reports the first demonstration of a pure vector beam with radial polarization achieved by the concept for the reduction of vortex phase. We also proposed a continuous body extended to the segmented non-axially-symmetric half-wave plate. Our technique enables the vector beam to be manipulated in singular optics. Our proposed concept can also be used in conjunction with generic technologies in other different fields, such as super resolution imaging, material science, multiplexing quantum information science, high energy physics, and even astronomy.

## Methods

### Internal Fresnel reflections

Internal Fresnel reflections are an example of fundamental optical phenomena that result in phase retardance of $\varphi =4{\tan }^{-1}\{\sqrt{{n}^{2}(\lambda ){\sin }^{2}\beta -1}\,/\,n(\lambda )\sin \,\beta \,\tan \,\beta \}$ between orthogonal polarizations, where *n*(*λ*) and *β* denote the refractive index of the material and the slope angle of a rhomb, respectively. Under the proposed conditions, *β* is a constant, while *n*(*λ*) shows dependence on wavelength *λ*. An output beam generates the phase retardance of *ϕ* by total internal reflections^35^.

### Non-axially-symmetric half–wave plate

A non-axially-symmetric half–wave plate, which possesses phase retardance of *ϕ* = π, is required for a generation of a pure vector beam with radial polarization and a uniform phase. A fast axis of the non-axially-symmetric half-wave plate is rotated with azimuth of *θ*/2 as a function of angle *θ*. In this experiment, we fabricated the non-axially-symmetric half-wave plate with four Fresnel rhomb segments. (see the subsections “Jones calculus” in the Methods section and Supplementary Note 1).

### Jones calculus

**Figure 8:**
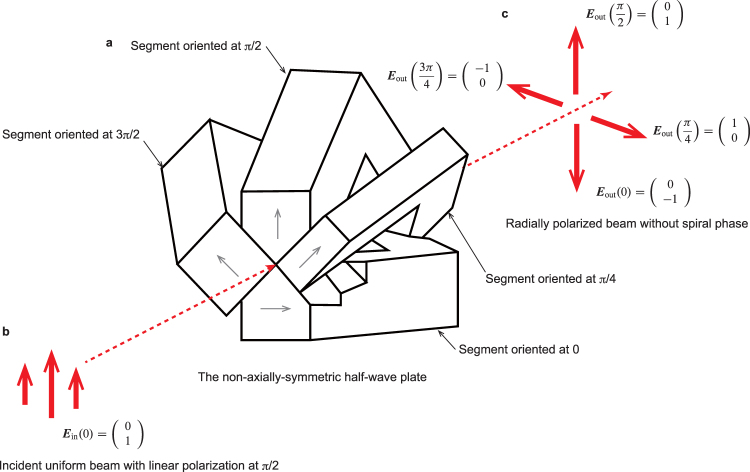
Conversion of vector beam by use of a non-axially-symmetric half-wave plate. (**a**) A non-axially-symmetric half–wave plate consists of four segments oriented at azimuth angles of 0, π/4, π/2, and 3π/4 radians. A segment is formed similar to a pair of Fresnel rhombs, resulting in internal reflections at slope angles. Based on four internal reflections, the relative relationship between orthogonal polarizations produces a phase retardance of π. (**b**,**c**) When an incident beam with Jones vector (0, 1)^T^ (i.e. linearly polarized at π/2 rad), the polarization after passing through segments oriented at 0, π/4, π/2, and 3π/4 rad can be determined as Jones vectors of (0, −1)^T^, (1, 0)^T^, (0, 1)^T^, and (−1, 0)^T^ via the use of Jones calculus. The vector beam is radially polarized with uniform phase.

When a uniform beam with linear polarization is incident onto a non-axially-symmetric half-wave plate that is acting as a radial polarization convertor (see Fig. 8a), the polarization of the vector beam can be calculated via Jones calculus^36–38^ to evaluate the spatial distribution of polarization and its phase in the vector beam. The Jones vector of the linear polarization at π/2 is described by ***E***_in_ = (0, 1)^T^ as shown in Fig. 8b. For calculation between the Jones vector and Jones matrix, the polarization of the vector beam, which is converted from the non-axially-symmetric half–wave plate fabricated in four parts, can be obtained in Fig. 8c (for more information, refer Supplementary Note 1).

### Experimental setup

**Figure 9:**
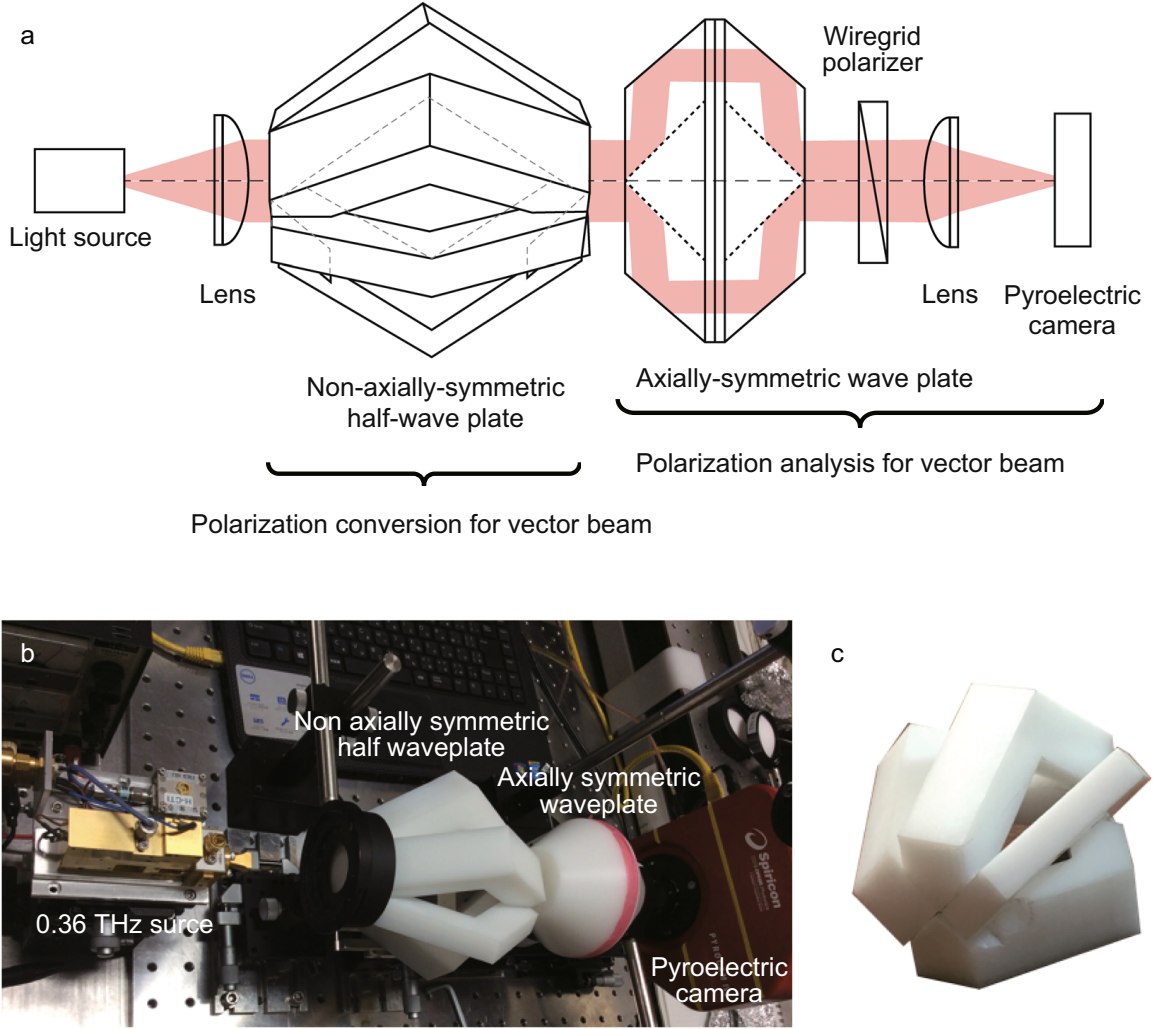
Experimental setup for polarization conversion and polarization analysis of vector beams. (**a**,**b**) A schematic drawing and a picture of experimental setup. (**c**) A picture of the non-axially-symmetric half-wave plate. A polarization conversion sets up consisting of a light source, a lens, and a non-axially-symmetric half-wave plate. The light source is a THz source at a frequency of 0.36 THz. A THz beam is linearly polarized at π/2. A vertically polarized beam with Stokes parameters of (1, −1, 0, 0)^T^ is converted into a vector beam with spatial distribution of Stokes parameters (*s*_0_(*θ*), *s*_1_(*θ*), *s*_2_(*θ*), *s*_3_(*θ*))^T^. The polarization of the vector beam generated by the polarization conversion is determined by polarization analysis through the use of an axially-symmetric wave plate, a wire-grid polarizer set at 0, and a pyroelectric array camera.

Figure 9a and b show a schematic drawing and a picture of the experimental setup for polarization conversion and polarization analysis of vector beams. The polarization conversion for the vector beams uses a THz light source, a THz lens (lens diameter *d* = 30 mm and focus length *f* = 100 mm), and the non-axially-symmetric half-wave plate with four segments. Figure 9c shows a picture of a non-axially-symmetric half-wave plate. The polarization analysis for the vector beams consists of an axially-symmetric wave plate, a wire-grid polarizer, a THz lens, and a pyroelectric array camera (Pyrocam IV Beam Profiling Camera, Ophir Optronics Solutions Ltd.; elements: 320 × 320, and pixel size: 80 µm) See Supplementary Note 3.

### Single-shot determination of polarization on vector beams

Spatial distribution of Stokes parameters *s*_0_(*θ*), *s*_1_(*θ*), *s*_2_(*θ*), and *s*_3_(*θ*) along the angle *θ* on the input vector beam converted are modulated by the axially-symmetric wave plate. After passing through a wire-grid polarizer, the intensity distribution of the vector beam varied as a function of the angle *θ* can be expressed as

1 $$I(\theta )={s}_{{\rm{C}}0}(\theta )=\frac{1}{2}{s}_{{\rm{A}}0}(\theta )+\frac{1}{4}{s}_{{\rm{A}}1}(\theta )-\frac{1}{2}{s}_{{\rm{A}}3}(\theta )\sin \,2\theta +\frac{1}{4}{s}_{{\rm{A}}1}(\theta ){\rm{cos4}}\theta +\frac{1}{4}{s}_{{\rm{A}}2}(\theta )\sin \,4\theta .$$

The intensity distribution *I*(*θ*) contains full Stokes parameters from *s*_A0_(*θ*) to *s*_A3_(*θ*) given in angular frequency terms — bias, sin 2*θ*, cos 4*θ*, and sin 4*θ*. To isolate each term in frequency space, we introduce Fourier transform method, which is commonly used for fringe pattern analysis in optical metrology^39,40^. Using Fourier transform method, Fourier spectrum of *P*(*k*) is expressed as

2 $$P(k)={ {\mathcal F} }^{-1}[I(\theta )]={C}_{0}(k)+{C}_{2}(k-{k}_{2})+{{C}_{2}}^{\ast }(k+{k}_{2})+{C}_{4}(k-{k}_{4})+{{C}_{4}}^{\ast }(k+{k}_{4}),$$

where *k* denotes angular frequency; ${ {\mathcal F} }^{-1}$ represents an inverse Fourier transform operator; and *C*_0_(*k*), *C*_2_(*k*), and *C*_4_(*k*) represent Fourier spectra; a superscript asterisk “^*^” represents a complex conjugate. By individually extracting the *C*_0_(*k*), *C*_2_(*k*), and *C*_4_(*k*) components in Fourier space, the spatial distribution of Stokes parameter*s s*_A0_(*θ*) − *s*_A3_(*θ*) are determined as

3a $${s}_{{\rm{A}}0}(\theta )=\frac{1}{2}(4\{ {\mathcal F} [{C}_{0}(k)]-{s}_{{\rm{A1}}}(\theta )\}),$$

3b $${s}_{{\rm{A}}1}(\theta )=8\Re \{ {\mathcal F} [{C}_{4}(k)]\},$$

3c $${s}_{{\rm{A}}2}(\theta )=8\Im \{ {\mathcal F} [{C}_{4}(k)]\},$$

3d $${s}_{{\rm{A}}3}(\theta )=4\Im \{ {\mathcal F} [{C}_{2}(k)]\},$$

where ${\mathcal F}$, $\Re$, and $\Im$ are the Fourier transform operator and the operators to extract the real and imaginary parts of their argument, respectively. For the spatial distribution of Stokes parameters, we determine the normalized Stokes parameters, ellipticity, and azimuth angle, respectively^38^ (for more information, refer to Supplementary Note 4).

### Determination of the geometric phase

To evaluate the phase distribution of the generated vector beam, we introduce geometric phase using the spatial distribution of Stokes parameters. The geometric phase was first suggested by Berry and Pancharatnam^33,41^, and it is related to cyclic changes of polarization on a Poincaré sphere with azimuth order of $\ell$ = 0. Here, we can estimate the geometric phase using the changes of the polarization represented by the Stokes parameters of (1, −1, 0, 0)^T^, (1, cos2*θ* cos2*ε*, sin2*θ* cos2*ε*, sin2*ε*)^T^, and (1, cos2*θ* cos2*ε*, sin2*θ* cos2*ε*, 0)^T^, where *ε* is ellipticity and *θ* is azimuth, respectively. The surface area *Ω* of a spherical triangle is expressed as ${\Omega }({C})=\int {\int }_{C}\sin \,\theta d\varepsilon d\theta ,$ where *C* is a domain of definition (for more information, refer Supplementary Note 5).

## Electronic supplementary material

supplementary

vectorbeam

magnitude
