# Supplementary material for: Demonstration of a terahertz pure vector beam by tailoring geometric phase
Source: Sci Rep. 2018 Jun 6;8:8690. doi: 10.1038/s41598-018-26964-7 (PMC5989212; doi:10.1038/s41598-018-26964-7)
Supplement: Supplementary file 1 — supplementary [file 41598_2018_26964_MOESM1_ESM.docx]

Supplementary

Demonstration of a terahertz pure vector beam by tailoring geometric phase

*Toshitaka Wakayama^1^, *Takeshi Higashiguchi^2,3^, Kazuyuki Sakaue^4,5^, Masakazu Washio^5^, and Yukitoshi Otani^3,6^

^1^School of Clinical Engineering, Faculty of Health and Medical Care, Saitama Medical University, Yamane 1397-1, Hidaka, Saitama 350-1241 Japan

^2^Department of Electrical and Electronic Engineering, Faculty of Engineering, Utsunomiya University, Yoto 7-1-2, Utsunomiya, Tochigi 321-8585, Japan

^3^Center for Optical Research & Education (CORE), Utsunomiya University, Yoto 7-1-2, Utsunomiya, Tochigi 321-8585 Japan

^4^Waseda Institute for Advanced Study, Waseda University, 3-4-1, Okubo, Shinjuku, Tokyo 169-8555 Japan

^5^Research Institute for Science and Engineering, Waseda University,

3-4-1, Okubo, Shinjuku, Tokyo 169-8555 Japan

^6^Department of Optical Engineering, Faculty of Engineering, Utsunomiya University, Yoto 7-1-2, Utsunomiya, Tochigi 321-8585, Japan

*Correspondence to [wakayama@saitama-med.ac.jp and higashi@cc.utsunomiya-u.ac.jp]"

**Supplementary Note 1 : Jones calculus**

When a uniform beam with linear polarization at π/2 is incident into a non-axially-symmetric half-wave plate as a radial polarization convertor shown in Supplementary Figure 1a, polarization of a generated vector beam can be calculated via a Jones vector and a Jones matrix [36-38] to evaluate spatial distribution of polarization and its phase in the vector beam. A relationship between the incident and output beams is presented as follows:

$E_{out}=JE_{in}$, (1)

where ***J***, ***E***_in_, and ***E***_out_ are the Jones matrix and the Jones vectors of the incident and output beams, respectively. The Jones vector of the linear incident polarization at π/2 is described by ***E***_in_ = (0, 1)^T^ as shown in Supplementary Figure 1b. The Jones matrix of the non-axially symmetric half-wave plate, which has a relative phase shift *φ* between the two orthogonal electrical field components *E_x_* and *E_y_*, is presented as follows:

. (2)

In the case of a conventional half waveplate with *φ* = π rad, the Jones matrix ***J***_HWP_ is simplified as follows:

$J_{HWP}=\left(\begin{matrix} 1 & 0 \\ 0 & -1 \end{matrix}\right)$. (3)

When a fast axis of the half waveplate is oriented at the angle *θ*, the Jones matrix ***J***_HWP_(*θ*) is rewritten using rotation matrix ***J***_R_:

$$J_{\text{HWP}}\left(\theta\right)=J_{\text{R}}\left(-\theta\right)J_{\text{HWP}}J_{\text{R}}\left(\theta\right)$$

$$=\left(\begin{matrix} \cos\theta& -\sin\theta\\ \sin\theta& \cos\theta\end{matrix}\right)\left(\begin{matrix} 1 & 0 \\ 0 & -1 \end{matrix}\right)\left(\begin{matrix} \cos\theta& \sin\theta\\ -\sin\theta& \cos\theta\end{matrix}\right)$$

$=\left(\begin{matrix} \cos2\theta& \sin2\theta\\ \sin2\theta& -\cos2\theta\end{matrix}\right)$. (4)

To calculate the Jones vector and Jones matrix, the polarization of the vector beam, which is converted from the non-axially-symmetric half-wave plate fabricated in four parts, can be obtained from Eqs. (1)–(4):

$E_{\text{out}}\left(0\right)=\left(\begin{matrix} 0 \\ -1 \end{matrix}\right)$, (5)

$E_{\text{out}}\left(\frac{\pi}{4}\right)=\left(\begin{matrix} 1 \\ 0 \end{matrix}\right)$, (6)

$E_{\text{out}}\left(\frac{\pi}{2}\right)=\left(\begin{matrix} 0 \\ 1 \end{matrix}\right)$, (7)

$E_{\text{out}}\left(\frac{3\pi}{4}\right)=\left(\begin{matrix} -1 \\ 0 \end{matrix}\right)$. (8)

The Jones vectors of Eqs. (5) and (7) represent vertical polarization at −π/2 and π/2, respectively, whereas the Jones vectors of Eqs. (6) and (8) represent the horizontal polarization at 0 and π, respectively. Therefore, the output polarization of the vector beam is radially polarized. This result corresponds to the Jones vector of the vector beam with radial polarization, which is described by (cos*θ*, sin*θ*)^T^. In addition to the polarization, we evaluated the vortex phase of the vector beam. Jones calculus can be used to determine the phase factor. When a uniform beam with linear polarization at π/2 is incident into the non-axially-symmetric half-wave plate, we obtain a pure vector beam with radial polarization and a uniform phase as shown in Supplementary Figure 1c because of a lack of phase factors of $\text{e}^{\text{i}\theta}$ in Eqs. (5)–(8). This result provides evidence for the reduction of vortex phase of $\text{e}^{\text{i}\theta}$ in the vector beam using the non-axially symmetric half waveplate as the radial polarization converter. Supplementary Note 2 shows the calculations between a Muller matrix and Stokes parameters for the non-axially-symmetric half-wave plate because the Stokes parameters is important for determination of geometric phase.

**Supplementary Note 2 : Calculation between Stokes vector and Mueller matrix**

Although Jones vectors are convenient for independently calculating polarization of a beam and its phase, the degree of polarization and partial polarization are difficult to represent via Jones vectors. To address this drawback, Stokes vectors containing Stokes parameters can be used to evaluate the beam. The Stokes parameters are obtained from the Mueller matrix ***M*** by methods of determining the polarization states described in Refs. [36-38]. A desired incident beam with a Stokes vector of ***S***_A_ and an output beam with a Stokes vector of ***S***_B_ are related to the Mueller matrix ***A***, which contains descriptions of polarization characteristics, such as birefringence, optical rotation, linear and circular dichroism. The relationship between the incident and output polarization is given by ***S***_B_ = ***AS***_A_. We can determine the Stokes vector ***S***_B_ when the Mueller matrix ***A*** and the Stokes vector ***S***_A_ are already known. The ***AS***_A_ is then used as the polarization generator. In conventional methods for determining the polarization states of the beam with a uniform polarization, we must consider only the uniform polarization states represented by ***S***_A_ = (*s*_A0_, *s*_A1_, *s*_A2_, *s*_A3_)^T^. However, to consider the polarization of the spatial distribution of polarization of vector beams, such as radially polarized beams, the spatial distribution of Stokes vectors on the vector beams containing a uniform beam with arbitrary polarization must be determined. The Stokes vectors of the vector beam can be expressed as ***S***(*θ*) = (*s*_A0_(*θ*), *s*_A1_(*θ*), *s*_A2_(*θ*), *s*_A3_(*θ*))^T^ at arbitrary angle of $\theta=\tan^{-1}\frac{y}{x}$, where *x* and *y* are the Cartesian coordinates in the *x*-*y* plane. To obtain a spatial distribution of Mueller matrix ***M***(*θ*), the conventional Mueller matrix is transformed by a rotation matrix ***R***(*θ*). The matrix ***M***(*θ*) is expressed as follows:

$$M\left(\theta\right)=R\left(\theta\right)MR\left(-\theta\right)$$

$=\left(\begin{matrix} 1 & 0 & 0 & 0 \\ 0 & \cos2\theta& -\sin2\theta& 0 \\ 0 & \sin2\theta& \cos2\theta& 0 \\ 0 & 0 & 0 & 1 \end{matrix}\right)\left(\begin{matrix} m_{00} & m_{01} & m_{02} & m_{03} \\ m_{10} & m_{11} & m_{12} & m_{13} \\ m_{20} & m_{21} & m_{22} & m_{23} \\ m_{30} & m_{31} & m_{32} & m_{33} \end{matrix}\right)\left(\begin{matrix} 1 & 0 & 0 & 0 \\ 0 & \cos2\theta& \sin2\theta& 0 \\ 0 & -\sin2\theta& \cos2\theta& 0 \\ 0 & 0 & 0 & 1 \end{matrix}\right)$. (9)

We herein consider the angular-variant Mueller matrix of an axially symmetric waveplate. The spatial distribution of Mueller matrix ***M***(*θ*) can be expressed by substituting the conventional Mueller matrix ***M*** into Eq. (9):

, (10)

where the conventional Mueller matrix ***M*** of a linear retarder with the uniform retardance *φ* and a fast axis oriented at *θ*  = 0 rad is given as , as shown in Refs. [36-38]. For example, if the waveplate has *φ* = π/2 and π, then the waveplate becomes an axially-symmetric quarter- and half-wave plate, respectively. The Mueller matrices of the axially symmetric quarter- and half-wave plate are given by substituting *φ* = π/2 and π into Eq. (10):

$M_{QWP}\left(\theta\right)=\left(\begin{matrix} 1 & 0 & 0 & 0 \\ 0 & \cos^{2}2\theta& \sin2\theta\cos2\theta& -\sin2\theta\\ 0 & \sin2\theta\cos2\theta& \sin^{2}2\theta& \cos2\theta\\ 0 & \sin2\theta& -\cos2\theta& 0 \end{matrix}\right)$, (11)

$M_{HWP}\left(\theta\right)=\left(\begin{matrix} 1 & 0 & 0 & 0 \\ 0 & \cos4\theta& \sin4\theta& 0 \\ 0 & \sin4\theta& -\cos4\theta& 0 \\ 0 & 0 & 0 & 0 \end{matrix}\right)$, (12)

where ***M***_QWP_(*θ*) and ***M***_HWP_(*θ*) are the Mueller matrices of the axially-symmetric quarter-wave plate and half-wave plate, respectively.

We consider that the non-axially-symmetric half-wave plate with four segments generated the vector beam with radial polarization and a uniform phase. In this case, the fast axis of the non-axially symmetric half waveplate is oriented by the rotation ratio of *θ*/2 along the angle *θ*. According to the Mueller matrix, the Mueller matrix of the non-axially-symmetric half-wave plate is obtained as follows:

$M_{NHWP}\left(\theta\right)=\left(\begin{matrix} 1 & 0 & 0 & 0 \\ 0 & \cos2\theta& \sin2\theta& 0 \\ 0 & \sin2\theta& -\cos2\theta& 0 \\ 0 & 0 & 0 & 0 \end{matrix}\right)$. (13)

The spatial distribution of Stokes vector of the output beam can be determined via the calculus between the Mueller matrix and Stokes vectors. Consider the Stokes vectors of the incident beam with the uniform polarization of (1, 1, 0, 0)^T^. For the Mueller matrices of Eq. (13), the exiting Stokes vector of the pure vector beam can be expressed by (1, cos 2*θ*, sin 2*θ*, 0)^T^. In this experiment, we employ a radial polarization convertor consisting of the non-axially-symmetric half-wave plate. Consider the Stokes vectors of the incident beam with a uniform polarization of (1, -1, 0, 0)^T^. For the Mueller matrices of Eq. (13), the exiting Stokes vector is obtained by (1, -cos 2*θ*, -sin 2*θ*, 0)^T^. As the result, the output beam is converted into the spatial distribution of polarization of the vector beam represented by (1, -1, 0, 0)^T^, (1, 1, 0, 0)^T^, (1, -1, 0, 0)^T^, and (1, 1, 0, 0)^T^ at angles of *θ* = 0, π/2, π, and 3π/2, respectively; thus, the converted beam becomes a radially polarized beam as one of the pure vector beams.

Jones calculus is convenient for evaluating the phase because Stokes parameters cannot be used to directly calculate the phase of the vector beam. However, the phase of the vector beam cannot be experimentally determined using the conventional polarization analysis of the Jones calculus without using interferometry. To overcome this drawback, we utilized the geometric phase caused by changes of the polarization on the Poincaré sphere (see Supplementary Note 5).

**Supplementary Note 3 : Experimental setup**

Supplementary Figure 2 shows an experimental setup for a polarization conversion and a polarization analysis of the vector beams. The polarization conversion for the vector beams used a THz light source, a THz lens (lens diameter *d* = 30 mm and focus length *f* = 100 mm), and a non-axially symmetric half waveplate with four segments. The polarization analyser for the vector beams consists of an axially symmetric waveplate, a wire-grid polarizer, a THz lens, and a pyroelectric array camera (Pyrocam IV Beam Profiling Camera, Ophir Optronics Solutions Ltd.; elements: 320 × 320, and pixel size: 80 µm). For the conversion into a vector beam, the uniform beam linearly polarized at π/2 is incident into the non-axially-symmetric half-wave plate. According to our Jones calculus for the vector beams, a pure vector beam with radial polarization is obtained by the conversion from a linearly polarized beam. To evaluate the spatial distribution of polarization of the vector beam, we first demonstrated how the spatial distribution of Stokes parameters of the vector beam is determined from a THz image as shown in Supplementary Figure 2. We also check the spatial distribution of polarization via ellipsometry using a traditional rotating analyser method (see the subsection Rotating analyser method in the Supplementary Note 6).

**Supplementary Note 4 : Single-shot determination of polarization on vector beams**

To spatially determine Stokes parameters on the vector beams via an axially symmetric waveplate from a THz image only, we constructed an experimental setup (Supplementary Figure 2), which consists of the components for the polarization conversion and the polarization analysis of the vector beams. The non-axially-symmetric half-wave plate is employed for the polarization conversion from an incident beam with uniform polarization to the vector beam with spatial distribution of Stokes parameters. The polarization analysis setup for the vector beam consists of an axially symmetric waveplate, a wire-grid polarizer, a THz lens, and a pyroelectric array camera (Pyrocam IV Beam Profiling Camera, Ophir Optronics Solutions Ltd.; elements: 320 × 320, and pixel size: 80 μm). For a THz light source, we used a Gunn diode (*f* = 0.36 THz, Continuous Wave Operation), which generates a uniform beam with linear polarization at π/2. The Stokes parameters of the vector beam converted by the non-axially symmetric half waveplate were modulated by the axially symmetric waveplate. After passing through the wire-grid polarizer, the intensity distribution of the vector beam varied as a function of the angle *θ*. Using a fast Fourier transform, the Fourier amplitude spectrum contains the spatial distribution of Stokes parameters *s*_0_(*θ*), *s*_1_(*θ*), *s*_2_(*θ*), and *s*_3_(*θ*). Each amplitude spectrum is filtered and separated in Fourier space. The Stokes parameters are reconstructed using an inverse fast Fourier transform. According to this method, the Stokes parameters can be spatially determined from a single-shot THz image. By using Stokes parameters on the Poincaré sphere, a geometric phase of the vector beam can be spatially determined.

To determine the spatial distribution of Stokes parameters of the vector beams, we employ the axially symmetric waveplate with spatial distribution of Mueller matrix, which is a 4 × 4 matrix that changes with respect to the angle *θ*. The Stokes vectors and the Muller matrix are obtained as follows:

$S_{C}\left(\theta\right)=X\left(\theta\right)S_{A}\left(\theta\right)$, (14)

$\left(\begin{matrix} s_{\text{C0}}\left(\theta\right) \\ s_{\text{C1}}\left(\theta\right) \\ s_{\text{C2}}\left(\theta\right) \\ s_{\text{C3}}\left(\theta\right) \end{matrix}\right)=\left(\begin{matrix} m_{00}\left(\theta\right) & m_{01}\left(\theta\right) & m_{02}\left(\theta\right) & m_{03}\left(\theta\right) \\ m_{10}\left(\theta\right) & m_{11}\left(\theta\right) & m_{12}\left(\theta\right) & m_{13}\left(\theta\right) \\ m_{20}\left(\theta\right) & m_{21}\left(\theta\right) & m_{22}\left(\theta\right) & m_{23}\left(\theta\right) \\ m_{30}\left(\theta\right) & m_{31}\left(\theta\right) & m_{32}\left(\theta\right) & m_{33}\left(\theta\right) \end{matrix}\right)\left(\begin{matrix} s_{\text{A0}}\left(\theta\right) \\ s_{\text{A1}}\left(\theta\right) \\ s_{A2}\left(\theta\right) \\ s_{A3}\left(\theta\right) \end{matrix}\right)$, (15)

where ***X***(*θ*) = ***AM***(*θ*). The combined Mueller matrix ***X***(*θ*) consists of the spatial distribution of Mueller matrix ***M***(*θ*) because of the axially symmetric waveplate indicated in Eq. (10) and the conventional Mueller matrix ***A*** for a linear polarizer set at a transmitting angle *θ* = 0. The Muller matrix ***A*** is expressed as follows:

$A=\frac{1}{2}\left(\begin{matrix} 1 & 1 & 0 & 0 \\ 1 & 1 & 0 & 0 \\ 0 & 0 & 0 & 0 \\ 0 & 0 & 0 & 0 \end{matrix}\right)$. (16)

The Mueller matrix ***A*** is not the spatial distribution of Mueller matrix but rather is the conventional Mueller matrix [36,38]. Substituting Eqs. (10) and (16) into Eq. (14), the spatial distribution of Stokes vector ***S*_C_** in Eq. (14) is determined. To simplify the calculations, the retardance *φ  *of the Mueller matrix ***A*** is set to *φ* = π/2. Using the Mueller matrix calculus, the intensity distribution *I*(*θ*) is defined as *s*_C0_(*θ*) in Eq. (15). For the relationship between the Mueller matrix and the Stokes vectors, the total intensity *s*_C0_(*θ*) is expressed as follows:

$S_{\text{C}0}\left(\theta\right)=\sum_{i=0}^{3} m_{0i}\left(\theta\right) s_{\text{A}i}\left(\theta\right)$. (17)

The intensity distribution can be then expressed as follows:

$I\left(\theta\right)=I_{\text{C}0}\left(\theta\right)=\frac{1}{2}s_{\text{A}0}\left(\theta\right)+\frac{1}{4}s_{\text{A}1}\left(\theta\right)-\frac{1}{4}s_{\text{A}3}\left(\theta\right)\sin2\theta+\frac{1}{4}s_{\text{A}1}\left(\theta\right)\cos4\theta+\frac{1}{4}s_{\text{A}2}\left(\theta\right)\sin4\theta$. (18)

The intensity distribution *I*(*θ*) contains the full Stokes parameters from *s*_A0_(*θ*) to *s*_A3_(*θ*) given in terms of bias, sin 2*θ*, cos 4*θ*, and sin 4*θ*.

To individually separate each term in frequency space, we introduce the Fourier transform method, which is commonly used for fringe pattern analyses in optical metrology [39,40]. To simplify the calculations in the Fourier analysis, the intensity distribution indicated in Eq. (18) is rewritten using the Euler formula of $\text{e}^{\text{i}\theta}=\cos\theta+\text{i}\sin\theta$ as follows:

$I\left(\theta\right)=c_{0}\left(\theta\right)+c_{2}\left(\theta\right)+c_{2}^{*}\left(\theta\right)+c_{4}\left(\theta\right)+c_{4}^{*}\left(\theta\right)$, (19)

with

$c_{0}\left(\theta\right)=\frac{2s_{A0}\left(\theta\right)+s_{\text{A}1}\left(\theta\right)}{4}$, (20)

$c_{2}\left(\theta\right)=\frac{\text{i}s_{A3}\left(\theta\right)}{4}\text{e}^{\text{i}2\theta}$, (21)

$c_{4}\left(\theta\right)=\frac{s_{\text{A}1}\left(\theta\right)-\text{i}s_{A2}\left(\theta\right)}{4}\text{e}^{\text{i}4\theta}$, (22)

where the asterisk “^*^” represents a complex conjugate. Considering the nature of the Fourier transform, the spatial distribution of Stokes parameters can be individually obtained by shifting followed by separating the real and imaginary parts. By shifting the Fourier spectra *c*_2_(*θ*) and *c*_4_(*θ*) to the origin in Fourier space, we are able to remove the phase factors of $\text{e}^{\text{i2}\theta}$ and $\text{e}^{\text{i4}\theta}$. By obtaining the real and imaginary parts of each Fourier spectrum, the spatial distribution of Stokes parameters can be determined. Using the inverse Fourier transform, Fourier spectrum of *P*(*k*) is expressed as follows:

$P\left(k\right)=F^{-1}\left[I\left(\theta\right)\right]=C_{0}\left(k\right)+C_{2}\left(k-k_{2}\right)+C_{2}^{*}\left(k+k_{2}\right)+C_{4}\left(k-k_{4}\right)+C_{4}^{*}\left(k+k_{4}\right)$, (23)

where *k* denotes the angular frequency; $F^{-1}$ represents the inverse Fourier transform operator; and *C*_0_(*k*), *C*_2_(*k*), and *C*_4_(*k*) represent Fourier spectra *c*_0_(*θ*), *c*_2_(*θ*), and *c*_4_(*θ*), respectively, which were obtained by calculating components from Eqs. (20)−(22). Such a Fourier spectrum contains the spatial distribution of Stokes parameters of the vector beam. The five Fourier spectra of *P*(*k*) centred at *k* = 0, ±*k*_2_, and ±*k*_4_ are independent over the frequency axis *k*. By individually extracting the *C*_0_(*k*), *C*_2_(*k*), and *C*_4_(*k*) components in Fourier space, the spatial distribution of Stokes parameters *s*_A0_(*θ*)-*s*_A3_(*θ*) are determined by Fourier transformations:

$s_{\text{A}0}\left(\theta\right)=\frac{1}{2}\left(4\left\{F\left[C_{0}\left(k\right)\right]-s_{\text{A}1}\left(\theta\right)\right\}\right)$ (24)

$s_{\text{A}1}\left(\theta\right)=8\Re\left\{F\left[C_{4}\left(k\right)\right]\right\}$ (25)

$s_{\text{A}2}\left(\theta\right)=8\Im\left\{F\left[C_{4}\left(k\right)\right]\right\}$ (26)

$s_{\text{A}3}\left(\theta\right)=4\Im\left\{F\left[C_{2}\left(k\right)\right]\right\}$ (27)

where $F$, $\Re$, and $\Im$ are Fourier transform operators and the real and imaginary parts of the Fourier transform, respectively. Eqs. (24)−(27) can be used to determine all the Stokes parameters from *s*_0_(*θ*) to *s*_3_(*θ*) containing the uniform arbitrary polarized beam.

Stokes parameters can specify the total polarization as well as the partial polarization. The relationship between the total and partial polarization is expressed as follows:

$s_{\text{A}}=\left(\begin{matrix} s_{\text{A}0}\left(\theta\right) \\ s_{\text{A}1}\left(\theta\right) \\ s_{\text{A}2}\left(\theta\right) \\ s_{\text{A}3}\left(\theta\right) \end{matrix}\right)=\left(1-P\right)\left(\begin{matrix} s_{\text{A}0}\left(\theta\right) \\ 0 \\ 0 \\ 0 \end{matrix}\right)+\left(\begin{matrix} s_{\text{A}0}^{'}\left(\theta\right) \\ s_{\text{A}1}^{'}\left(\theta\right) \\ s_{\text{A}2}^{'}\left(\theta\right) \\ s_{\text{A}3}^{'}\left(\theta\right) \end{matrix}\right)$ , (28)

where P is the degree of polarization, and it is determined as follows:

$P=\frac{\sqrt{s_{\text{A}1}^{2}\left(\theta\right)+s_{\text{A}2}^{2}\left(\theta\right)+s_{\text{A}3}^{2}\left(\theta\right)}}{s_{\text{A}0}\left(\theta\right)}$ (29)

For complete polarization and natural polarization, the degrees of polarization become P = 1 and P = 0, respectively. In the case of partial polarization, the degree of polarization is described by 0 < P < 1. We also evaluate the ellipsometric parameters, such as the ellipticity *e* and its azimuth *ϕ*, which are expressed as follows:

, (30)

. (31)

Further details regarding the degree of the polarization, ellipticity and its azimuth are described in Ref. [38].

**Supplementary Note 5 : Determination of the geometric phase**

To evaluate phase distribution of a generated vector beam, we introduce the geometric phase using a spatial distribution of Stokes parameters. The geometric phase was suggested by Berry and Pancharatnam [33,41], and it is related to the cyclic changes of the polarization on a Poincaré sphere with  = 0. Supplementary Figure 3 shows the Poincaré sphere with the order of  = 0. The equator on the Poincaré sphere represents the linear polarization with different azimuthal angles. The north and south poles represents right and left circular polarizations. The point indicated by “A” on a Poincaré sphere describes the (1, -1, 0, 0)^T^ polarization of the beam generated from the light source in Supplementary Figure 3a. The conventional quarter waveplate generates the right circular polarization shown by “B” on the Poincaré sphere. Using the axially symmetric quarter waveplate, the spatial distribution of polarization of the generated vector beams is illustrated for points “C” and “D”. The geometric phase obtained by the spatial distribution of polarization can be determined from circuit *C* of a spherical triangle ABC and ABD, where A, B, C, and D are located at (1, -1, 0, 0)^T^, (1, 0, 0, 1)^T^, (1, 0, -1, 0)^T^, and (1, 1, 0, 0)^T^, respectively. For example, the enclosed surface areas ABC and ABD become *Ω*(*C*) = π/2 and *Ω*(*C*) = π, respectively. When the non-axially-symmetric half-wave plate is used, the changes of the polarization on the Poincaré sphere are illustrated in Supplementary Figure 3b. In this case, the enclosed surface area is not drawn on the Poincaré sphere. Therefore, the geometric phase is zero, which indicates that the vortex phase in the vector beam can be reduced. Here, we can estimate the geometric phase using the changes of the polarization represented by the Stokes parameters (1, -1, 0, 0)^T^, (1, cos2*θ *cos2*ε*, sin2*θ *cos2*ε*, sin2*ε*)^T^, and (1, cos2*θ *cos2*ε*, sin2*θ *cos2*ε*, 0)^T^. The surface area *Ω* of a spherical triangle is expressed as follows:

$\Omega\left(\theta\right)=\iint_{C} \sin\theta d\varepsilon d\theta$. (32)

The total geometric phase is found to be $\gamma\left(C\right)=\Omega\left(C\right)/2$, where *C* is the domain of definition. Based on the spatial distribution of polarization and the geometric phase of the vector beam, whether the generated vector beam is a radially polarized beam that reduces the vortex phase can be determined. As mentioned above, this process can be used to determine the polarization states of the vector beams with higher order Stokes parameters. However, we also conclude that determining short pulse polarization is limited by the thickness of the axially symmetric waveplate.

**Supplementary Note 6 : Rotating analyser method**

A rotating analyser method of THz ellipsometry using a wire-grid polarizer for the THz region is implemented to evaluate spatial distribution of polarization of an incident beam with a uniform polarization from the THz source. For the two-dimensional polarization analysis, we used 36 sampling images.

To evaluate the spatial distribution of polarization of the incident beam from the THz source for validation, we employed ellipsometry based on the rotating analyser method. Substituting Eq. (16) into Eq. (9), the Mueller matrix of a linear polarizer with transmitting angle *θ* is obtained as follows:

$B=\frac{1}{2}\left(\begin{matrix} 1 & \cos2\theta& \sin2\theta& 0 \\ \cos2\theta& \cos^{2}2\theta& \sin2\theta\cos2\theta& 0 \\ \sin2\theta& \sin2\theta\cos2\theta& \sin^{2}2\theta& 0 \\ 0 & 0 & 0 & 0 \end{matrix}\right)$. (33)

From Eqs. (15) and (33), we obtained the intensity distribution to evaluate the polarization of the incident beam from the light source.

$I\left(\theta\right)=\frac{1}{2}s_{0}+\frac{1}{2}s_{1}\cos2\theta+\frac{1}{2}s_{2}\sin2\theta$. (34)

The rotating analyser method is not sensitive to the Stokes parameter *s*_3_ component because the rotating analyser method is used with a linear polarizer. To obtain the three components of the intensity distribution *s*_0_, *s*_1_, and *s*_2_, we captured four images with the transparent angle of the wire-grid polarizer set at 0, π/4, π/2, and 3π/4, respectively.

$I\left(0\right)=\frac{1}{2}s_{0}+\frac{1}{2}s_{1}$, (35a)

$I\left(\frac{\pi}{4}\right)=\frac{1}{2}s_{0}+\frac{1}{2}s_{2}$, (35b)

$I\left(\frac{\pi}{2}\right)=\frac{1}{2}s_{0}-\frac{1}{2}s_{1}$, (35c)

$I\left(\frac{3\pi}{4}\right)=\frac{1}{2}s_{0}-\frac{1}{2}s_{2}$. (35d)

For the four images *I*(0), *I*(π/4), *I*(π/2), and *I*(3π/4), the Stokes parameters *s*_0_, *s*_1_, and *s*_2_ can be obtained as follows:

$s_{0}=\frac{I\left(0\right)+I\left(\text{π}/4\right)+I\left(\text{π}/2\right)+I\left(3\text{π}/4\right)}{2}$ (36a)

$s_{1}=I\left(0\right)-I\left(\text{π}/2\right)$ (36b)

$s_{2}=I\left(\text{π}/4\right)-I\left(3\text{π}/4\right)$ (36c)

We assume that the incident beam is completely polarized because of $s_{0}^{2}=s_{1}^{2}+s_{2}^{2}+s_{3}^{2}$. As a result, the ellipticity *e* is expressed as follows:

$e=\frac{1-\sqrt{s_{1}^{2}+s_{2}^{2}}}{1+\sqrt{s_{1}^{2}+s_{2}^{2}}}$. (37)

We note that the azimuth of the ellipical polarization is determined by Eq. (31) because the *s*_3_ component of the Stokes parameters is not used.

To evaluate the spatial distribution of polarization of the generated vector beam, we also employed the abovementioned ellipsometry based on the rotating analyser method to validate our results. In this experiment, the axially symmetric waveplate was removed in the optical configuration of the polarization analysis for the vector beam. Supplementary Figure 4 shows the intensity distribution captured by the pyroelectric array camera with the rotating polarizer at 0 (Supplementary Figure 4a), π/4 (Supplementary Figure 4b), π/2 (Supplementary Figure 4c), and π (Supplementary Figure 4d). For the ellipsometry measurements, we show the intensity distribution *s*_0_ (Supplementary Figure 4e) and azimuth *ϕ* (Supplementary Figure 4g). A comparison of the intensity distribution *s*_0_ described in Supplementary Figure 4e and the intensity distribution determined from Eq. (24) in Supplementary Figure 4f indicates that the distributions are consistent. The azimuth of the elliptical polarization is radially distributed on the beam. This comparison shows that the vector beam generated from the non-axially-symmetric half-wave plate beam becomes the pure vector beam with radial polarization and our proposed technique can be used for the single-shot determination of the spatial distribution of Stokes parameters of the vector beam.

**Supplementary Note 7 : Polarization behaviour of the vector beam in free space**

To reproduce propagation behaviour of a generated vector beam in a vacuum and in air, we numerically simulated the complex behaviour of the vector beam from the polarization and its phase. As shown in Supplementary Figure 5, we characterized the beam propagation of the converted beam by changing the propagator *ωt* – *kz* from 0 to 7π/4, where *ω*, *t*, *k*, and *z* are angular frequency, time, wave number, and direction, respectively. A detailed description (Supplementary Figures 6a–6h) of the polarization dynamics is provided in Supplementary Movies 1 and 2, which are time-sequential movies of the dynamics for the vector beam in a short frame interval of π/4 rad. The cross-sectional beam profile in Supplementary Figure 6a was reconstructed from the two-dimensional distribution of the polarization and the phase. Their magnitudes in Supplementary Figure 6a are also normalized by the intensity distribution. Most electric fields were axial-symmetrically vibrating on the angular-variant vector beam. Moreover, we show the magnitudes of the vector beam obtained from the intensity distribution in Supplementary Figure 6a. The changes of the electric field are shown in Supplementary Figures 7a–7h. Compared with the polarization and its phase in Fig. 1c, the propagation behaviour of the vector beam with radial polarization in Supplementary Figure 5 corresponded to the output polarization and the phase of “B” in Fig. 1c. Thus, we realized a pure vector beam with radial polarization reducing the vortex phase.

**Supplementary Figure 1** : **Conversion of the vector beam by use of the non-axially-symmetric half-wave plate.** **a**, Non-axially-symmetric half-wave plate consists of four segments oriented at 0, π/4, π/2, and 3π/4. The segment is formed similar to a pair of rhomb’s, which results in internal reflections at slope angles. Based on four internal reflections, relative relationship between orthogonal polarizations produces phase retardance of π. **b, c,** When the incident beam indicated by the Jones vector of (0, 1)^T^ is linearly polarized at π/2 rad, the polarization after passing through the segments oriented at 0, π/4, π/2, and 3π/4 rad can be determined as Jones vectors of (0, -1)^T^, (1, 0)^T^, (0, 1)^T^, and (-1, 0)^T^ via the use of Jones calculus. The vector beam is radially polarized with a uniform phase.

**Supplementary Figure 2 : Experimental setup for polarization conversion and polarization analysis of vector beams.** The polarization conversion setup consists of a light source, a lens, and a non-axially-symmetric half-wave plate. The light source is used as a THz source at frequency of 0.36 THz. The THz beam is linearly polarized at π/2. A vertically polarized beam with Stokes parameters of (1, -1, 0, 0)^T^ was converted into a vector beam with spatial distribution of Stokes parameters (*s*_0_(*θ*), *s*_1_(*θ*), *s*_2_(*θ*), *s*_3_(*θ*))^T^. The polarization of the vector beam generated by the polarization conversion is determined by the polarization analysis and conducted by an axially symmetric waveplate, a wire-grid polarizer set at 0, and a pyroelectric array camera.

**Supplementary Figure 3 : Geometric phase obtained from the** **Poincaré sphere.** Geometric phase can be determined by calculating enclosed surface area drawn by changes of polarization on a Poincaré sphere. The geometric phase can be used to evaluate the phase of a generated vector beam. Here, we show a comparison of the enclosed area determined by changes in the polarization on a Poincaré sphere versus the area determined by changes in the combinations between (**a**) the incident circular polarization and the axially-symmetric quarter-wave plate and between (**b**) the incident linear polarization and non-axially-symmetric half-wave plate. **a.** Changes of the polarization are indicated by the enclosed area of “ABC” and “ABD”. The geometric phases yielded by the enclosed areas “ABC” and “ABD” become *γ*(*C*) = π/4 and π/2, respectively, because the enclosed areas of these are illustrated by *Ω*(*C*) = π/2 and π, respectively. **b.** In the case of the non-axially-symmetric half-wave plate, the geometric phase is zero because the changes of the polarization states shown by arcs “EF” and “EG” do not indicate the enclosed area on the Poincaré sphere.

**Supplementary Figure 4** : **Experimental results for the ellipsometry type of the rotating analyser method.** **a–d.** Images captured by the pyroelectric array camera are shown after passing through the wire-grid polarizer set at 0, π/4, π/2, and 3π/4. The arrows in subfigures a-d describe the transmitting angle of the polarizer. **e.** Intensity distribution of the vector beam is reconstructed from four images. **f.** Intensity distribution from Eq. (24). A comparison of Figs. 4e and 4f shows that these images are consistent. **g.** Azimuth of the polarization ellipse. The azimuth is distributed as the function of the angle *θ*. The results obtained from the rotating analyser method can be used for validation.

**Supplementary Figure 5** : **Propagation behaviour of the vector beam in three-dimensional free space.** The vector beam is changed by the propagator *ωt* – *kz* from 0 to 7π/4. Arrows indicate the electric field of the vector beam. The magnitudes of the electric field are normalized by the intensity distribution. A detailed description is provided at a short frame interval of π/4 rad.

**Supplementary Figure 6** : **Vector map for electric field of the vector beam.** **a – h.** Detailed description of the polarization dynamics is provided in Supplementary Movie 1, which is a time-sequential movie of the dynamics for the radially polarized beam at a short frame interval of π/4 rad.

**Supplementary Figure 7** : **Two-dimensional distribution of electric field on the vector beam.** **a – h.** Magnitudes of radially polarized beam obtained from the intensity distribution in Supplementary Figure 6a. Changes of the electric field are shown in Supplementary Figures 7a-7h. A detailed description is provided in Supplementary Movie 2 at a short frame interval of π/4 rad.
